# Neuronal (Bi)Polarity as a Self-Organized Process Enhanced by Growing Membrane

**DOI:** 10.1371/journal.pone.0024190

**Published:** 2011-09-14

**Authors:** Silvia A. Menchón, Annette Gärtner, Pablo Román, Carlos G. Dotti

**Affiliations:** 1 Department of Molecular and Developmental Genetics, VIB and Center for Human Genetics, Katholieke Universiteit Leuven (KULeuven), Leuven, Belgium; 2 Department of Mathematics, Katholieke Universiteit Leuven (KULeuven), Leuven, Belgium; 3 Centro de Biología Molecular Severo Ochoa, CSIC-UAM, Madrid, Spain; University of Michigan, United States of America

## Abstract

Early *in vitro* and recent *in vivo* studies demonstrated that neuronal polarization occurs by the sequential formation of two oppositely located neurites. This early bipolar phenotype is of crucial relevance in brain organization, determining neuronal migration and brain layering. It is currently considered that the place of formation of the first neurite is dictated by extrinsic cues, through the induction of localized changes in membrane and cytoskeleton dynamics leading to deformation of the cells' curvature followed by the growth of a cylindrical extension (neurite). It is unknown if the appearance of the second neurite at the opposite pole, thus the formation of a bipolar cell axis and capacity to undergo migration, is defined by the growth at the first place, therefore intrinsic, or requires external determinants. We addressed this question by using a mathematical model based on the induction of dynamic changes in one pole of a round cell. The model anticipates that a second area of growth can spontaneously form at the opposite pole. Hence, through mathematical modeling we prove that neuronal bipolar axis of growth can be due to an intrinsic mechanism.

## Introduction

**Figure 1:**
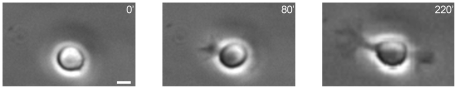
Establishment of bipolar cell axis in hippocampal neurons. Development of an individual hippocampal neuron grown *in vitro* was followed by time lapse microscopy. Scale bar 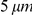, (neurons in similar developmental stages immunolabeled with a neuron-specific antibody are shown in Fig. S1).

During development many cellular processes depend on the highly polarized distribution of molecules on the cell membrane. The ability of cells to acquire and maintain a morphological asymmetry involves localized cytoskeletal changes and polarized membrane traffic. The generation and maintenance of polarity are very important for many complex biological activities. Neurons are among the cell types with the most prominent asymmetry, by establishing dendritic vs. axonal domains which are different in function and morphology. The correct establishment of polarized domains in neurons enables their directional migration and polarized axon-dendrite formation and is thus one of the most critical steps in brain development. Neuronal polarization starts with the selection of the site from which the first neurite will grow [1] before morphological changes are evident [1], [2]. Recently, we demonstrated that the second neurite forms opposite to the first, not randomly (Fig. 1, [3]). This has important consequences for neuronal development, since like that initial polarity axis determines the axis of migration and defines axonal and dendritic domains [4].

The site from which the first neurite emerges is defined by the localized accumulation or activation of molecules with the capacity to directly or indirectly induce a local deformation in the plasma membrane. Even before the morphological deformation occurs, newborn neurons display polarized exo- and endocytosis and cytoskeletal rearrangements [1], [3]. Polarized growth however can be induced by the action of cues inherited from the past division (G. Pollarolo and C.G. Dotti personal communication). However, it is not clear whether the formation of the second neurite also requires “external” triggering mechanisms or is the consequence of a “passive” mechanism, derived from the first one, similar to the trailing edge of a motile cell, which only requires the determination of a ligand-induced leading edge.

To test this hypothesis we used a mathematical model. We based our model on those proposed by Altschuler *et al.* [5] and Turing [6]. Our model assumes an activator-inhibitor dynamics and diffusion-driven instabilities. Different from other models describing polarity establishment [5], [7]–[11], we include membrane growth. We study spontaneous symmetry breaking and how polarity domains are affected by membrane growth. To experimentally validate predictions of our model we compare results of our simulations with intensity distributions of Sec8. Sec8 is a exocyst subunit localized in multiple endocytic compartments. Its intensity is a measure of endocytic and exocytic traffic which is correlated with protein accumulation on the plasma membrane due to dynamic maintenance. Our approach suggests that localized membrane growth enhances polarity and it predicts second bud localization.

## Results

### The Model

Before the formation of the first neurite, a polarized distribution of molecules has to be generated. This can occur by different mechanisms in a tight temporal sequence. One such mechanism is asymmetric endocytosis and exocytosis, which normally play an important role in maintaining a dynamic equilibrium of protein concentrations on the cell membrane through constant recycling [12]. Asymmetric endo-exocytosis triggers changes in lateral diffusion, which can contribute to a further increase in the asymmetry or to its stabilization. Membrane protein asymmetry can also be achieved through localized changes in cytoskeleton dynamics, which not only regulate mechanical forces but also control the formation of membrane extensions and local protein concentration. In fact, the polarized distribution of membrane proteins, including receptors, transporters and adhesion molecules is due to such dynamic asymmetries. However, in order to exert asymmetric function, these proteins require the contribution of different types of functional “adaptors”, such as lipids, scaffolding proteins, small GTPases and kinases and phosphatases.

**Figure 2:**
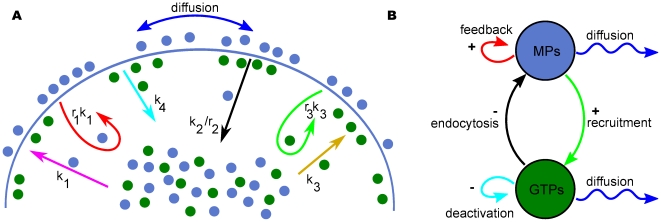
Schematic representation of our model. (A) Blue circles represent membrane proteins and green circles represent modulators of endocytosis. Membrane proteins and modulators of endocytosis can diffuse along the cell membrane. Arrows indicate biological events: magenta, spontaneous membrane association; red, positive feedback; black, endocytosis; ocher, spontaneous activation; cyan, deactivation; green, activation through recruitment; blue, lateral diffusion for membrane proteins and modulators of endocytosis. The solid line represents the cell membrane (total length, 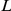). (B) activator-inhibitor scheme. In our model, membrane proteins and modulators of endocytosis play the roles of activator and inhibitor, respectively. For polarity domain formation modulators of endocytosis have to diffuse faster than membrane proteins.

We formulated a model assuming two variables of asymmetric distribution: traffic to and from the plasma membrane and lateral diffusion. We considered two different model molecules, the first, named 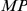, represents a typical integral membrane protein endocytosed by a canonical clathrin-mediated process (e.g., cadherin); and the second one, named 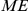, representing a modulator of 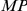-endocytosis (e.g., p120-catenin). Therefore, we supposed that 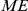 regulates internalization of 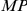 and considered the following biological events, (shown schematically in Fig. 2A):

1. *Spontaneous membrane association:* Membrane proteins are tethered spontaneously to the cell membrane [13], [14]. We consider it occurs with a constant rate 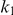.
2. *Membrane association through recruitment:* A positive feedback circuit recruits membrane proteins to the places in which they are already localized [5], [15]. The characteristic rate of this process is considered proportional to the amount of membrane proteins at that place with a proportionality constant 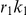, (in this work, membrane association through recruitment is also referred as positive feedback).
3. *Endocytosis:* The endocytosis pathway is regulated by the amount of modulators of endocytosis [16], [17]. In our model the endocytosis rate is proportional to the concentration of modulators of endocytosis, with a constant 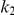, and follows a Michaelis-Menten kinetic for membrane proteins with a maximum carrying capacity: 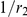.
4. *Spontaneous activation:* Modulators of endocytosis are activated spontaneously [18]. We assume this happens with a constant rate 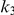.
5. *Deactivation:* Modulators of endocytosis can be deactivated [18]. We consider a deactivation rate proportional to its concentration with 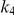 as proportionality constant.
6. *Activation through recruitment:* In order to regulate the concentration of proteins on the cell membrane, the activation of modulators of endocytosis is also induced by membrane proteins [19]. In our model this occurs with a rate proportional to membrane protein concentration and a proportionality constant 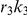.
7. *Lateral Diffusion:* membrane proteins and modulators of endocytosis diffuse on the cell membrane. Diffusion coefficients are correlated with molecule size being smaller (slow diffusion) for larger particles [20].

These rules were mainly based on dynamic trafficking membrane, being exocytosis (membrane addition) represented by 1 and 2, while 3 reflects endocytosis whose dynamics is regulated through 4–6. To differentiate between “membrane association through recruitment”, (point 2), and “activation through recruitment”, (point 6), we called them *positive feedback* and *recruitment*, respectively. The first three biological events regulate the dynamic temporal variation of the membrane protein concentration on the cell membrane, while the following three drive the dynamic temporal variation of the modulator of endocytosis concentration on the cell membrane. We represented these rules by a classic scheme for an activator-inhibitor system [15], [21] (Fig. 2B). In these systems the activator induces its own production as well as the production of the inhibitor; and the inhibitor inhibits both production (here, production describes processes which increase the local availability of molecules). In our model, the activator is represented by membrane proteins and the inhibitor is characterized by modulators of endocytosis.

### Mathematical Formulation and Analysis of the nonlinear system

Our model can be written as the following partial differential equation system:
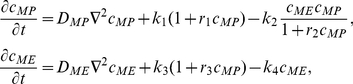
(1)where 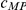 and 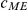 are the concentrations on the cell membrane of membrane proteins and modulators of endocytosis, respectively; and 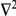 represents the Laplace-Beltrami operator. The upper equation in Eq. (1) indicates the temporal evolution of the membrane protein concentration. On the right-hand side, the first term corresponds to diffusion of membrane protein with diffusion coefficient 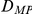, the second term has contributions due to spontaneous membrane association and positive feedback, and the last term describes endocytosis with a Michaelis-Menten kinetics for 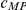. The lower equation in Eq. (1) describes the temporal evolution of the concentration of modulators of endocytosis. On the right-hand side, the first term corresponds to diffusion of modulators of endocytosis with diffusion coefficient 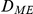, the second term represents the spontaneous activation and the activation through recruitment, and the last term represents the inhibitor deactivation.

This system has Turing-instabilities if the homogeneous equilibrium point is stable in the absence of spatial variation but unstable to *small* perturbations if diffusion is present. Since neuronal polarity does occur in two dimensions reflected by the fact that it occurs in cell adhered to a substratum *in vitro*, we chose to work using a two-dimensional system. In order to have a complete analysis, it is convenient to work with a dimensionless system. Defining 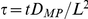, with 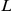 the characteristic length of the system, 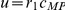 and 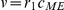 we arrived to the following non-dimensional system
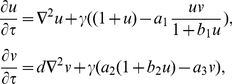
(2)where 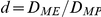, 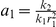, 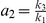, 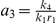, 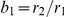, 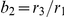 and 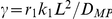. The parameter 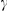 is defined as the ratio of membrane protein diffusion characteristic time to the positive feedback characteristic time and 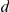 is the ratio of the diffusion coefficient of modulators of endocytosis to the diffusion coefficient of membrane proteins. By definition 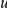 and 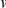 should be non-negative numbers. Defining the following functions
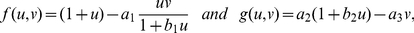
(3)our system can be written as:
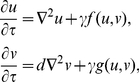
(4)which is equivalent to the system (2.7) presented in Murray [21]. From now on, 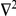 represents the non-dimensional Laplace-Beltrami operator. Choosing polar coordinates 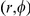 for the spatial distribution and using the transformation 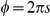; 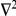 becomes 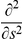, with 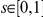, (we consider 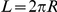 with 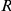 the cell radius).

Relevant homogeneous steady states 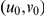 of Eq. (2) are positive solutions of 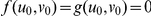. These equilibrium values reflect a balance between the production and the loss of membrane proteins and modulators of endocytosis. In other words, equilibrium points satisfy:
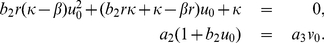
(5)where 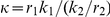, 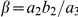 and 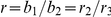. The parameter 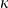 is proportional to the local positive feedback rate and inversely proportional to the maximum local endocytosis rate.

Instabilities due to diffusion should be spatially dependent and in the presence of non-spatial variation steady states should be stable. Proceeding as Murray [21], linear stability without considering spatial variation is guaranteed if
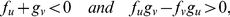
(6)where 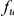, 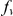, 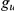 and 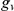 are the partial derivatives of 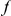 and 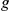 evaluated at the steady state. For our model:
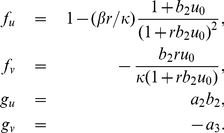
(7)On the other hand, diffusion-driven instabilities are present in presence of spatial variation if
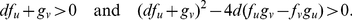
(8)Previous equations define a critical value 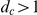. If the inhibitor diffuses more than 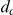 times faster than the activator (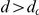), the system may have diffusion-driven instability. The wave-numbers of unstable modes, 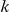
's, depend on the dynamics of 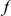 and 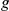, and the values of 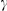 and 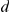 as well, (see Murray [21] for an extensive mathematical formulation). Our non-dimensional system can be described with seven parameters, 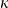, 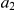, 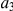, 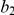, 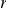, 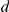 and 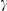, the first five are related with the interactions between membrane proteins and modulators of endocytosis and the last two are the only ones including diffusion.

Relevant steady states were calculated from Eq. (5). Depending on the system dynamic, we defined three different cases:

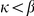
. Only one positive solution.

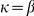
. Only one positive solution if 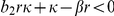.

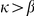
. Two positive solutions if 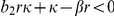 and 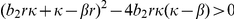.

**Figure 3:**
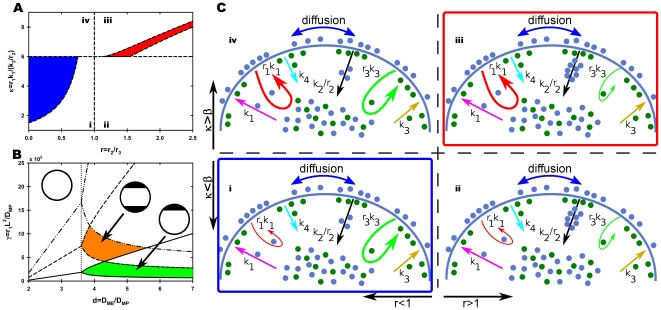
Phase and bifurcation diagrams for pattern formation. (A) Phase diagram for 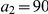, 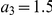 and 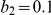. The blue and red shaded areas and the solid line represent the regions in which the system may form polarity domains. The final number of polarity domains depends on the relationship between 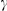 and 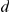. Bifurcation diagram for 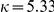 and 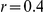 is shown in (B). In the region on the left of the dotted line, cells can not polarize. The green and orange shaded areas display conditions under which one or two polarity domains can be formed. (C) A schematic representation of the intensity of the biological processes involved, color code is the same as Fig. 1B. In regions ii and iii, black arrows have more particles attached indicating that endocytic vesicles saturate for smaller membrane protein concentration. Polarity domains can be formed only in the regions i and iii. These regions were remarked with a frame whose color was assigned according to the shaded areas in (A).

The relationship 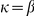 can be expressed as 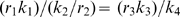, which indicates a balance between the production and loss rates of both kinds of molecules. The conditions expressed by Eq. (6) and Eq. (8) can be analyzed for each case. If 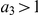 (i.e. 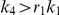) the first condition in Eq. (6) is guaranteed for all the cases; this defines a maximum for the local feedback rate. However, the second condition is never satisfied for the highest root in the case ( *c*). For fixed 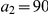, 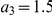 and 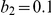 we drew a phase diagram 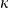 vs. 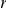 which is shown in Fig. 3A. The parameter 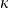 increases if the local feedback rate increases or if the maximum local endocytosis rate decreases. On the other hand, 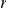 increases if 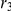 or 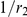 decrease, when 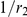 decreases the local endocytosis rate has higher values for the same local concentration of membrane proteins. Polarity domains can be formed in systems whose parameters are inside the shaded regions or on the solid line. The blue and red areas indicate the parameters which can generate patterns for the cases ( *a*) and ( *c*), respectively. The solid line represents the parameters which can generate polarity domains for the case ( *b*). Kinetic parameters in agreement with Eq. (6) and Eq. (8), which are represented en Fig. 3A, are needed but are not sufficient for polarity domain formation. The final pattern depends on the relationship between 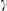 and 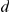. For each point in the shaded areas or on the solid line in Fig. 3A there is a diagram as the one that is shown in Fig. 3B for 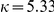 and 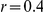. For 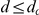 Turing instabilities are not present and polarity domains could not emerge as the time increases. For 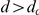 the number of polarity domains, 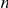, depends on the unstable mode wave-numbers. In Fig. 3B, the wave-number related with 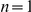 is unstable in the region between the solid lines; in the region between the dashed lines the unstable wavenumber is the one associated with 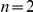, (the area between the dash-dotted lines represents the region with unstable wavenumber corresponding to 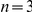). In the intersection area between these regions the final pattern depends on the dominant solution which is that with the highest eigenvalue (see Fig. S2). For simplicity, we only marked the regions with a single unstable wavenumber characterized by 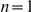 or 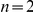, which are the green and orange shaded areas, respectively, in Fig. 3B, (see Fig. S3 for equivalent bifurcation diagrams).

### Conceptual Interpretation of Polarity Domain Formation

Our model assumes that polarity domains can be established by three basic mechanisms acting at the same time. First, an autocatalytic mechanism which is local and self-reinforcing and is due to variations in protein concentration on the membrane. The variations can be the consequence of an intrinsic fluctuation or the presence of asymmetric external cues. Although the cause of the variation is irrelevant for this model, a good example of localized protein variation in neurons is N-cadherin (G. Pollarolo and C.G. Dotti personal communication). Second, a local amplification of the concentration change through the production or recruitment of modulator of endocytosis (e.g. p120-catenin [22]). An example of this is illustrated by the increased concentration of endocytosis at the pole from which later the first neurite will grow [1]. Third, a long range inhibition via a slow-acting and fast-diffusing inhibitor, to ensure that inhibition occurs after activation so to maintain the local activating process in a confined region of the plasma membrane [15], [21]. In our model these conditions can be satisfied if modulators of endocytosis diffuse faster than membrane proteins, (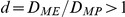), and if there is a balance between endocytosis and exocytosis, which can also be seen as a balance between the carrying capacity of endocytosis, (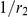), and the local concentration of membrane proteins which equalizes the local activation through recruitment rate to the local spontaneous activation rate, (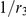). The phase diagram (Fig. 3A), shows that polarity domains can be formed if the parameters are inside the shaded regions or on the solid line. Therefore, polarized domains could emerge suddenly on the cell membrane after perturbations of the equilibrium state. In the non-shaded regions, very low local positive feedback rates are not sufficient to form an initial cluster of membrane proteins or very high local positive feedback rates enhance all the clusters on the entire cell membrane leading to an homogeneous state without polarization. Then, local perturbations vanish with the time and can not be stabilized. On the other hand, variations in 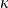 can also be due to variations in 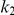. An increase in 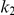 is correlated with a decrease in 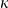 and a decrease in 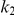 is correlated with an increase in 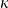. Thus, our model suggests that mutant phenotypes with a very low or very high endocytosis rates are not able to form polarity domains. Other kinds of mutant phenotypes may have different 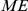 activation rates, 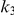, which can be seen as a variation in the parameter 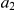. Our approach suggests that for higher 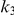, a higher feedback is needed in order to maintain polarity domains, (see Figs. S4 and S5). In Fig. 3C we show a schematic representation of the contribution of the involved biological processes in the four regions defined in (A). In regions i and iv endocytic vesicles saturate after the local activation through recruitment rate becomes higher than the local spontaneous activation rate. Therefore, membrane proteins stay longer on the cell membrane and lower local feedback rates are needed to generate polarity. On the other hand, in regions ii and iii endocytic vesicles saturate before the local activation through recruitment rate becomes higher than the local spontaneous activation rate. Thus, there is a higher internalization of membrane proteins and higher local feedback rates are needed to generate polarity. If endocytic vesicles saturate at a concentration around the concentration in which the local activation through recruitment rate becomes similar to the local spontaneous activation rate, (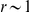), it is not possible to establish asymmetries for any feedback rate. The final polarized state and the number of polarity domains (caps) depend on the ratio of the diffusion coefficient of modulators of endocytosis to the diffusion coefficient of membrane proteins (Fig. 3B). If modulators of endocytosis diffuse slowly (on the left of the dotted line) cells can not polarize. Faster diffusing modulators of endocytosis allow the establishment of polarity domains and the number of them depends on the parameter 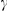, increasing for faster positive feedback. For a same value of 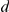, solutions with 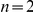 have a higher local feedback rate or less mobile membrane proteins than solutions with 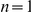.

### Symmetry breaking

Polarity domains can be established spontaneously even in a homogeneous environment suggesting that the cell can be seen as a self-organized system which can break the initial symmetry generating an asymmetric pattern from *small* fluctuations around the homogeneous state. We used our model to study the formation of stable polarity domains on the cell membrane from a quasi-uniform state. In particular, we solved the nonlinear system numerically considering an initial condition *near* the steady state given by 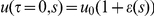 where 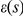 are random numbers between 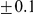; i.e., we selected as initial condition a random perturbation about the steady state value, 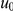, smaller than 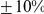.

Some of the parameters used in our simulations were estimated from experimental data. According to Michelson *et al.* and Jilkine *et al.* the effective total concentration of the modulator of endocytosis can be considered as 2000 nM [23], [24], being its 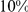 on the membrane [25]–[27]. The diffusion coefficient of the modulators of endocytosis and its rate of deactivation were set to 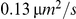 [28] and 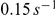 [5], respectively. On the other hand, the diffusion coefficient for membrane proteins and its concentration on equilibrium were assumed at values 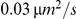 [29], [30] and 30 nM [31], respectively. The parameters used in the simulations were based on these data and taking into account that the size of a cell is around 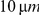 in diameter. Table 1 summarizes the values of the kinetics parameters which also correspond with our previous selection for Fig. 3.

**Table 1:** Numerical values of kinetics parameters.

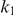	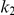	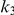	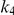	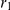	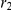	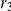
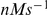	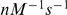	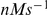	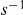	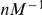	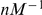	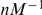
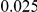	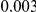	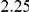	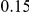	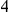	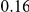	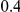

**Figure 4:**
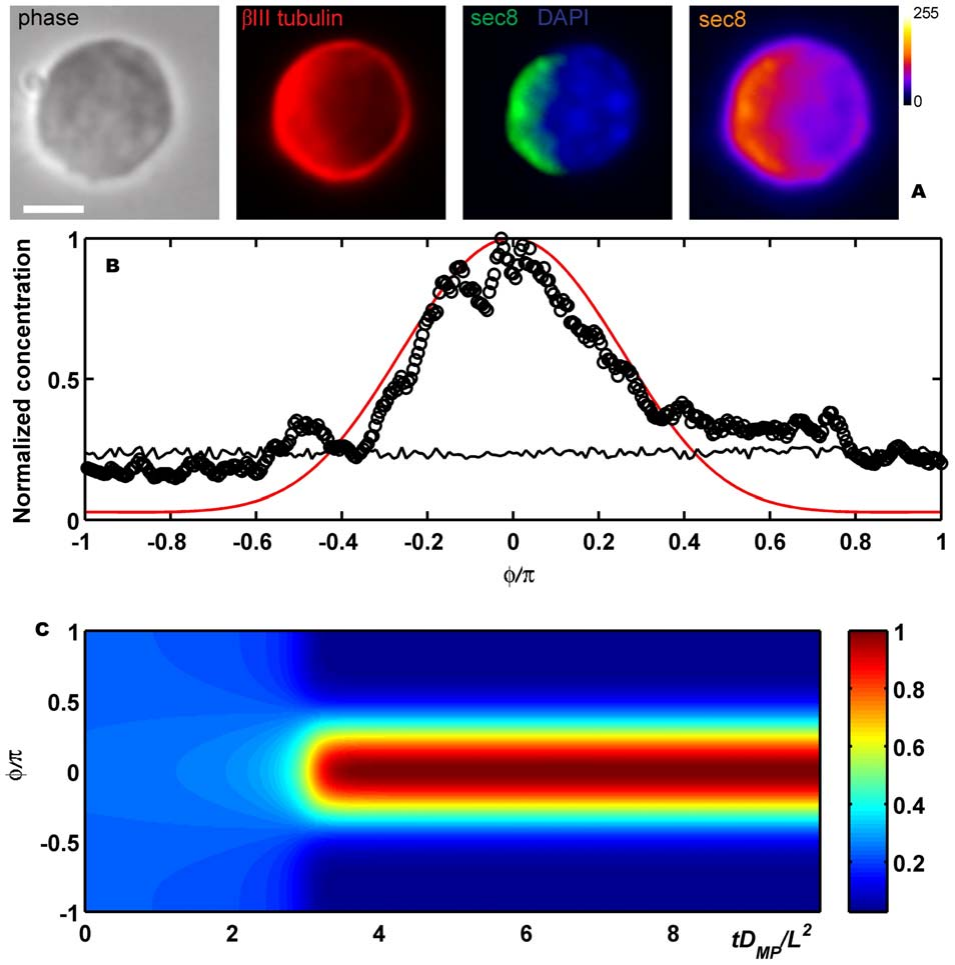
Symmetry breaking. (A) Hippocampal neurons were fixed shortly after plating and immunolabeled with a neuron-specific anti-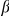 tubulin antibody (red), an anti Sec8 antibody (green) and a nuclear marker (blue). The right panel shows a pseudocolor image of the Sec8 only. Scale bar 5 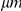. (B) Experimental results, Sec8, (open circles) compared with numerical simulations, 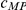, (red line) for the round neuron shown in the inset. Initial profile (random perturbations) in black. In this case 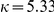, 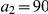, 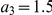, 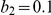, 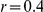, 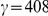 and 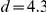. (C) Temporal evolution of the simulation shown in (B) (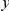 axis, membrane position; 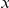 axis, time; color scale, normalized 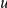 values). Results are normalized to the final maximum value of 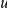.

In order to compare our simulation with experimental results we analyzed the distribution of the Sec8 subunit of the multiprotein exocyst complex. The exocyst is accumulated at sites which display high exo- and endocytosis rates and due to its vesicle-membrane tethering activity [32] it is important for the local accumulation of membrane proteins and the activation of modulators of endocytosis. Moreover the exocyst is important for polarized exocytosis [32] and membrane addition [33] and will therefore mark regions of polarizing domains which will lead to membrane expansion such as neurite growth [34] and to membrane turnover [35], which is also important in order to maintain polarized domains. We determined the intensity of Sec8 in morphological unpolarized round hippocampal neurons developing in an homogeneous environment and found that Sec8 concentrates at one maximum in agreement with simulations performed using our model (Figs. 4A and 4B). Although only one representative cell is shown, the majority of round neurons express this pattern of monopolar Sec8 accumulation, (see Fig. S6). A snapshot of the temporal evolution of the spatial patterns for 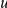 starting from a random configuration is shown in Fig. 4C. The uniform solution becomes unstable and Turing patterns appear remaining very stable as a state of dynamic equilibrium while time increases. From an initial random configuration, places where a stable maximum appears, are also random. However, since we used periodic boundary conditions, we chose the central position for the maximum in order to have a nicer plot.

### Pattern formation on a growing membrane

The previous analyses were performed for a system evolving on a circular non-growing membrane. However, we also wanted to analyze the system behavior while a neurite starts growing. In this case, the Laplace-Beltrami operator in Eq. (1) has to be modified for taking into account the effect of growing membrane and changes in geometry. In order to get the appropriate differential equation system we proceeded as Plaza *et al.* [36]. We represented the cell membrane growing by a one-dimensional function 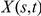. For describing a neurite growing we used the following explicit form:
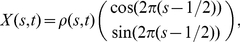
(9)with 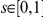 and
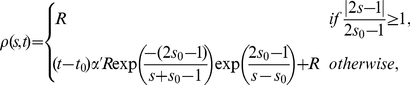
(10)where 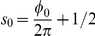, 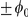 are the polar angles in which the bud starts and ends, 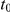 is the time in which the bud starts growing and 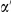 is the rate of growth which is considered constant. This function satisfies the required conditions by Plaza *et al.* [36]. A schematic plot of this curve is shown in Fig. S7 at three different times.

Defining the arc length function 
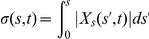
 and keeping the same notation as Plaza *et al.* [36], the reaction diffusion system in Eq. (1) on a growing membrane takes the following non-dimensional form
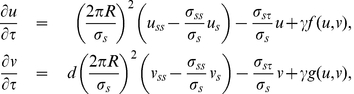
(11)where subindexes indicate partial derivative, (i.e. 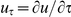, 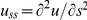), and the explicit form for 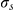, 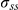 and 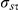 are given by:
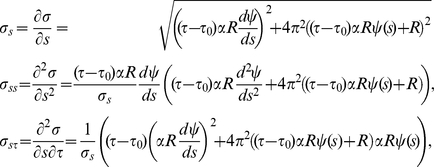
if 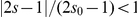, where 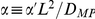 is the dimensionless parameter associated with 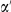 and
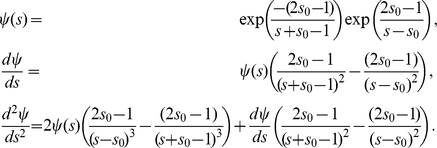
For 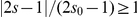 the explicit expressions are: 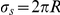, 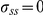 and 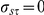. If there is no growing, the dimensionless parameter 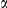 is equal to zero. Therefore, 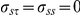 and 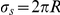 for all 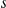, and as we expected, the system of Eq. (11) becomes the one in Eq. (4).

### Growing membrane and bipolarity

**Figure 5:**
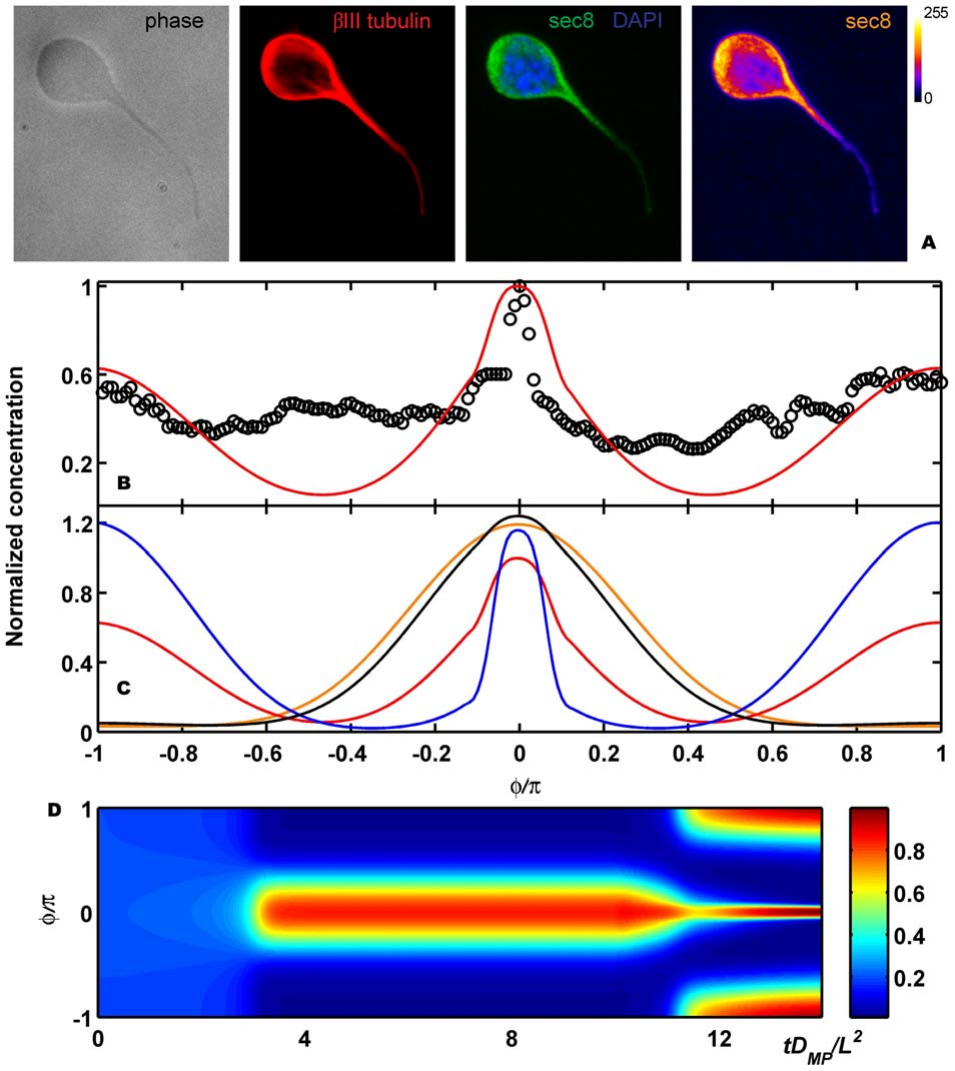
Polarity domain formation in neurons with one neurite. (A) Hippocampal neurons were fixed shortly after plating and immunolabeled with a neuron-specific anti-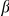 tubulin antibody (red), an anti Sec8 antibody (green) and a nuclear marker (blue). The right panel shows a pseudocolor image of Sec8 only. Scale bar 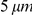. (B) Experimental results, Sec8, compared with numerical simulation, 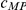. The normalized Sec8 intensity of a single neuron is represented by open circles. The base of the first neurite is located in the center (0 degree) of the graph. The red line shows the normalized numerical results for a membrane growing with speed 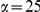 at 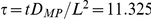. Initial profile (orange) as well as profiles at 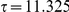 for different speeds (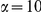, black; 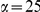, red; 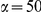, blue) are shown in (C). Profiles are normalized with respect to the maximum value of the red line. In (D) the temporal evolution of the normalized concentration of membrane proteins for a cell with a growing membrane is presented, growth starts at 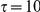 and 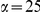, (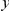 axis, angular membrane position; 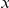 axis, time; color scale, normalized concentration values). For a dynamic representation see Movie S1. For all the simulations kinetics parameters are as in Fig. 4.

Irrespective of the mechanism by which an asymmetric accumulation of membrane proteins occurs, it activates a series of events, i.e. cytoskeletal changes, which result in the production of a localized membrane deformation followed by the generation of the cylindrical neurite. Therefore, a consequence of local molecular asymmetry is a change in the membrane geometry and curvature. Due to this, the lateral diffusion of molecules is modified. Since, the generation of the first neurite is followed by the appearance of a second one at the opposite pole, we next asked if the existence of localized growth in one pole had a influence on the establishment of a membrane asymmetry in the opposite pole. For this, we performed simulations considering a neurite growing as Eq. (9). We did not model the relationship between inhibitor/activator and cytoskeleton, we just took into account that the cell membrane starts growing at the place where the accumulation of membrane proteins is located. In order to perform our simulations, we proceeded as in Section “Symmetry Breaking” for 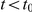 and we solved the Eq. (11) numerically for 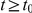. Thus, growth starts after the pattern with one maximum has become stable. In Fig. 5 the temporal evolution of a simulation and the profiles for different membrane growth speeds at the same time are shown. Immediately after starting growth, only one maximum is still present; which is thinner than the one at 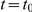. At later times, a stable pattern with two maxima appears. One maximum is located at the same place in which symmetry breaking occurred, but it is thinner, while the second is located at the opposite site. Thus, starting with a small perturbation from the homogeneous state a stable polarity domain can be formed. This polarity domain changes by adding a localized growth. The system evolves by reinforcing protein accumulation of particles at the place of growth and a second maximum is generated at the opposite side defining the axial orientation (see Movie S1). When the second maximum becomes stable its intensity is comparable with the intensity of the first one. At a given time, different profiles can be obtained depending on the growth speed which affects the relative strength between maxima. For a high speed of neurite outgrowth both maxima could already have a similar intensity, while for very slow growth the second maximum could have not appeared yet.

Experimental data, obtained by analyzing the membrane distribution of Sec8, in neurons with one neurite showed the presence of a second oppositely localized accumulation of Sec8. Its intensity for one sample neuron with one neurite is shown in Figs. 5A and 5B (open circles). As the model predicted (Fig. 5B, solid line), we found a maximal accumulation of Sec8 at the pole of the first bud growth but also a second maximum at the opposite site. Simulations and experiments are qualitatively in agreement.

## Discussion

In this work, we developed a mathematical model to analyze cell polarity considering dynamic traffic to and from the plasma membrane, positive feedback, diffusion, curvature and membrane growth. The model presented here can be considered as a conceptual model to study early stages of neuronal polarity. However, it can also be used to explain polarity in other cell types in which an interaction between dynamic recycling and exchange of membrane proteins and lateral diffusion are present.

Our approach was based on an activator-inhibitor system which includes a local self-reinforcing process and a global inhibition. Polarity domains arise from the interplay between activator-inhibitor when there is a dynamic balance between diffusion and membrane traffic, turning noise or local signals into asymmetries which remain stable in time. The local activating process, which is a positive feedback loop, is necessary for polarity domain formation. However, very high or low positive feedback rates lead to symmetric states. Our model defines optimal regions for positive feedback rates which mainly depends on internalization rate values (Fig. 3). We included asymmetric membrane growth to analyze how that would affect the next step in polarity establishment and maintenance. We induced a growing membrane at the place in which the symmetry breaking event had occurred. Our simulations indicated that the original accumulation becomes even more localized and it allows the generation of a new polarity domain at the side opposite to that of growth, identical to how it happens in “real life” [3]. As a matter of fact, bipolarity is an essential differentiation event *in vivo*, utilized first to assure proper radial migration of the young neuron and later to confer/fix axonal and dendritic properties to the, respectively, apical and basal neurites. Similar results can be obtained if growth starts at a place where an accumulation of proteins has been induced. We provide biological data showing that our simulations data are in total agreement with molecular organization and distribution in cells.

It is worth clarifying, that even if the neuron was treated as what it is, a three-dimensional object, still the phase and bifurcation diagrams would be equivalent. In three dimensions, the reaction terms are not modified. Considering a cell as a sphere without growing, the Eq. (4) is still valid, but with a different Laplacian operator that is convenient to be written in spherical coordinates. Since we are modeling the cell membrane, we are interested in the sphere surface for a given radio, 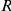. The eigenfunctions of the spherical Laplacian, (which are the functions we have to look at for describing the spatial distribution for the linear approximation), are the spherical harmonics, 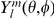, with eigenvalues 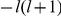, (while in two-dimensions the eigenfunctions of the Laplacian were 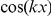 with eigenvalues 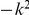). The phase diagram and all the analyses are similar to those in the two-dimension case considering now 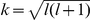 instead of 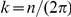 [21]. The spherical harmonics associated with 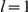, are the functions characterized by positive values in one hemisphere and negative values in the opposite. Since the scenarios are equivalent, we can speculate what will happen after adding growth. There is a scale change when the surface is expanded by allowing growth. Since the behavior in two- and three-dimensional systems without growing is the same, we can expect the same qualitative change due to an increase of the domain. The qualitative change is the shift to the phenotype characterized with the solution of the following eigenvalue. Making the bud growth in the center of the accumulation and considering the spherical harmonic associated with 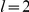, we hypothesize that the phenotype with more likelihood to appear is the one correlated with the spherical harmonic 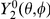, that is characterized by two maxima at the opposite poles.

Although our model is based on interactions between exocytosis, endocytosis and lateral diffusion, other participating events in cell morphogenesis, such as interactions between growth factor receptors and the cytoskeleton, have been neglected for simplicity and because they are, as shown here, not crucial to the effects we have described. In any case, the model presented here could be adapted to evaluate their influence in neurite outgrowth.

**Figure 6:**
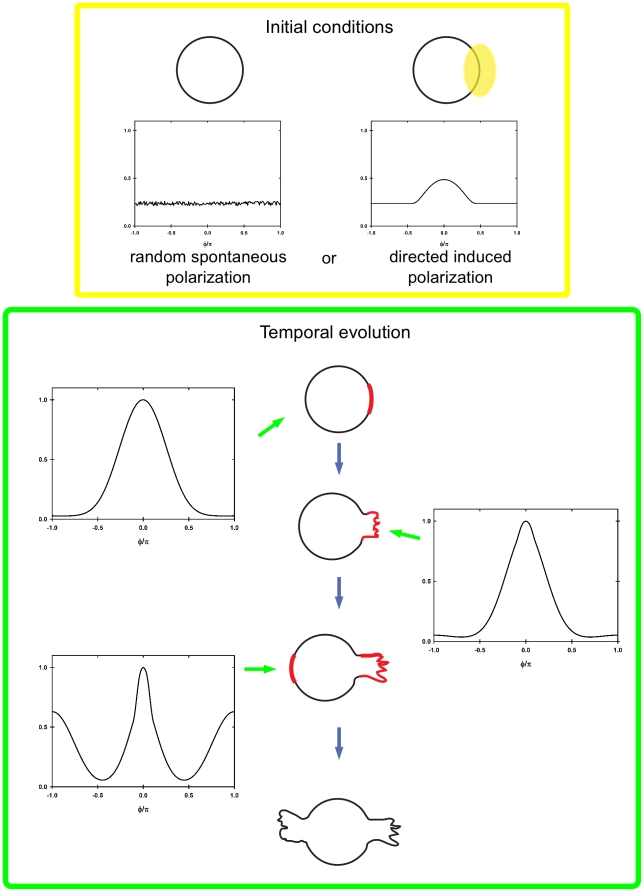
Schematic representation of neuronal (bi)polarity. Whether because of spontaneous or exogenous changes in the immediate post-mitotic neuron (initial conditions), a stable accumulation develops (red crescent, upper cell in temporal evolution panel). This maximum favors growth (middle cell), in turn favoring, the generation of a second maximum at the opposite pole and the occurrence of neuronal bipolar phenotype (lower cell). Representations of our model solutions are also shown at different stages.

In summary, our model is consistent with the following scenario: first, intrinsic or extrinsic determinants (eg. mitotic-inherited signal or cell-cell/matrix contact, respectively) induce a change in protein concentrations in a focal point of the plasma membrane. This change derives in the stabilization of the newly formed accumulation which can favor growth. Upon growth, a second accumulation appears spontaneously at the pole opposite to the site of growth, in turn leading to bipolar shape. Hence, in mathematical terms, bipolar neuronal morphology requires the occurrence of a single, monopolar, active event, responsible for the first deformation. Once this occurred, the second deformation takes place passively. Although we can not rule out that the second neurite may form *in vivo* through an active process, our results indicate that the second pole is, minimally, predisposed by the occurrence of the first. These changes, combined with our model solutions, are illustrated in Fig. 6. Hence, our mathematical approach captures the most important characteristics of neuronal polarity at the early stages, explaining that a localized membrane growth at the place where an intrinsic or extrinsic signal determined accumulation not only favors polarization but also predicts and determines that the second neurite would localize at the opposite site. This is exactly how cortical neurons start migration.

## Materials and Methods

### Numerical calculations

In order to construct numerical solutions to our model we approximate 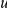 to a discrete solution defined in a one dimensional spatial grid of 200 points at a given discrete time, 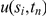. The solution has to be periodic in the space since we consider the cell as a circle. For simplicity we only show the discrete equations for 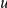. The Eq. (11) can be written considering different operators as
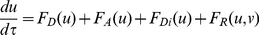
(12)where 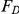, 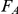, 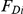 and 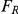 represent diffusion, advection, dilution and reaction operators, respectively. The expression for Eq (4) is similar, but advection and dilution operators are not present. The solution from 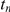 to 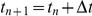 can be generated using splitting operator methods. In order to avoid spurious modes we choose the temporal step according to stability criteria [37]–[39]. For the spatial derivative we use a first-order approximation and for the time derivative the explicit Euler discretization.

### Primary cultures

Rat embryonic hippocampal neurons were prepared [40] and plated at a density of 2,500 cells per 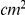 on poly-L-lysine (PLL) coated coverslips.

### Immunocytochemistry

Neurons were fixed after 1 to 5 hours with 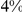 PFA (with 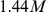 sucrose, 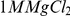, 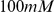 EGTA) at 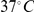 for 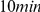. Cells were permeabilized for 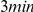 in 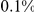 Triton X-100/PBS. After blocking in 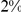 FBS, 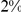 BSA, and 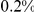 fish gelatine in PBS, neurons were incubated with the primary antibody for 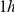 at room temperature or at 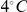 overnight. Secondary Alexa conjugated antibodies (Invitrogen) were added for 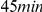 after washing in PBS.

The following primary antibodies were used: anti Sec8 (kind gift from Shu-Chan Hsu, Rutgers University, NJ) and anti 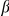 III-Tubulin from rabbit (Covance).

### Quantification of Sec8

Quantification of the intensity of membrane Sec8-labeling was performed using the open source ImageJ software (Rasband, W.S., ImageJ, NIH, USA). Neurons were identified by the neuron-specific marker 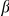 III-tubulin. A band with constant pixel width along the perimeter of the neuron was selected and radial sums of fluorescent intensities were measured. Data were plotted according the angle position of the radial line. The neurite itself was not considered in the analysis.

### Ethics Statement

All animal experiments were approved by the Ethics Committee of the K.U.Leuven, Biosafety and Biotechnology authorization number AMV/17122007/SBB219.2007/0341.

## Supporting Information

Figure S1
**Neuron developmental stages.** Hippocampal neurons were fixed at different times after plating and immunolabeled with the neuron-specific anti-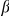 tubulin antibody (red, upper three panels) or, after longer differentiation time (lower panel) with an antibody which is specific for a dendritic protein (Map2 in red) and one specific for an axonal protein (anti Tau-1 in green).(TIFF)

Figure S2
**Unstable eigenvalues.** The eigenvalue, 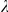, for the solution obtained linearizing Eq. (4) about the steady state 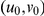 versus 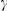 for 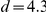, (A), and 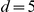, (B). Solid, dashed and dash-dotted lines represent eigenvalues for the unstable modes 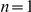, 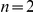 and 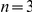, respectively. In the regions where more than one unstable mode is present, the final number of polarity domains is defined by the highest one. For this figure 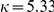, 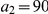, 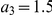, 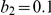 and 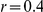.(TIFF)

Figure S3
**Bifurcation diagrams (I).** Different bifurcation diagrams for a system characterized by Fig. 3A. On the left, 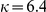 and 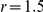; and on the right 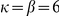 and 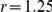.(TIFF)

Figure S4
**Phase and bifurcation diagrams (II).** A phase diagram for a mutant phenotype with a lower modulator of endocytosis activation rate is shown in the upper left corner. Bifurcation diagrams for the indicated values are also shown. In this picture, 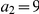, 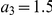 and 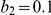, (thus, 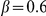).(TIFF)

Figure S5
**Phase and bifurcation diagrams.** A phase diagram for a mutant phenotype with a higher modulator of endocytosis activation rate is shown in the upper left corner. Bifurcation diagrams for the indicated values are also shown. In this picture, 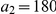, 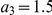 and 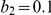, (thus, 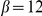).(TIFF)

Figure S6
**Quantification of Sec8 accumulation.** Hippocampal neurons were fixed shortly after plating and immunolabeled with a neuron-specific anti-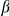 tubulin antibody and an anti Sec8 antibody. The fluorescence of Sec8 along the membrane was quantified. The signal was normalized and maxima of different cells aligned with each other respect the quarter with the highest intensity. The curve shows the mean value of 38 round neurons and the inset the analysis of the mean fluorescence of each quarter.(TIFF)

Figure S7
**Growing domain.** The curves represent the cell surface with a growing bud between the polar angles 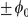. At 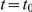 the cell boundary is a perfect circle.(TIFF)

Movie S1 **Polarity domain formation and membrane growth.** Dynamic representation of the membrane protein concentration shown in Fig. 5D, (color scale, normalize concentration values).
(AVI)
