# Supplementary figures and images for: Neuronal (Bi)Polarity as a Self-Organized Process Enhanced by Growing Membrane
Source: PLoS One. 2011 Sep 14;6(9):e24190. doi: 10.1371/journal.pone.0024190 (PMC3173449; doi:10.1371/journal.pone.0024190)

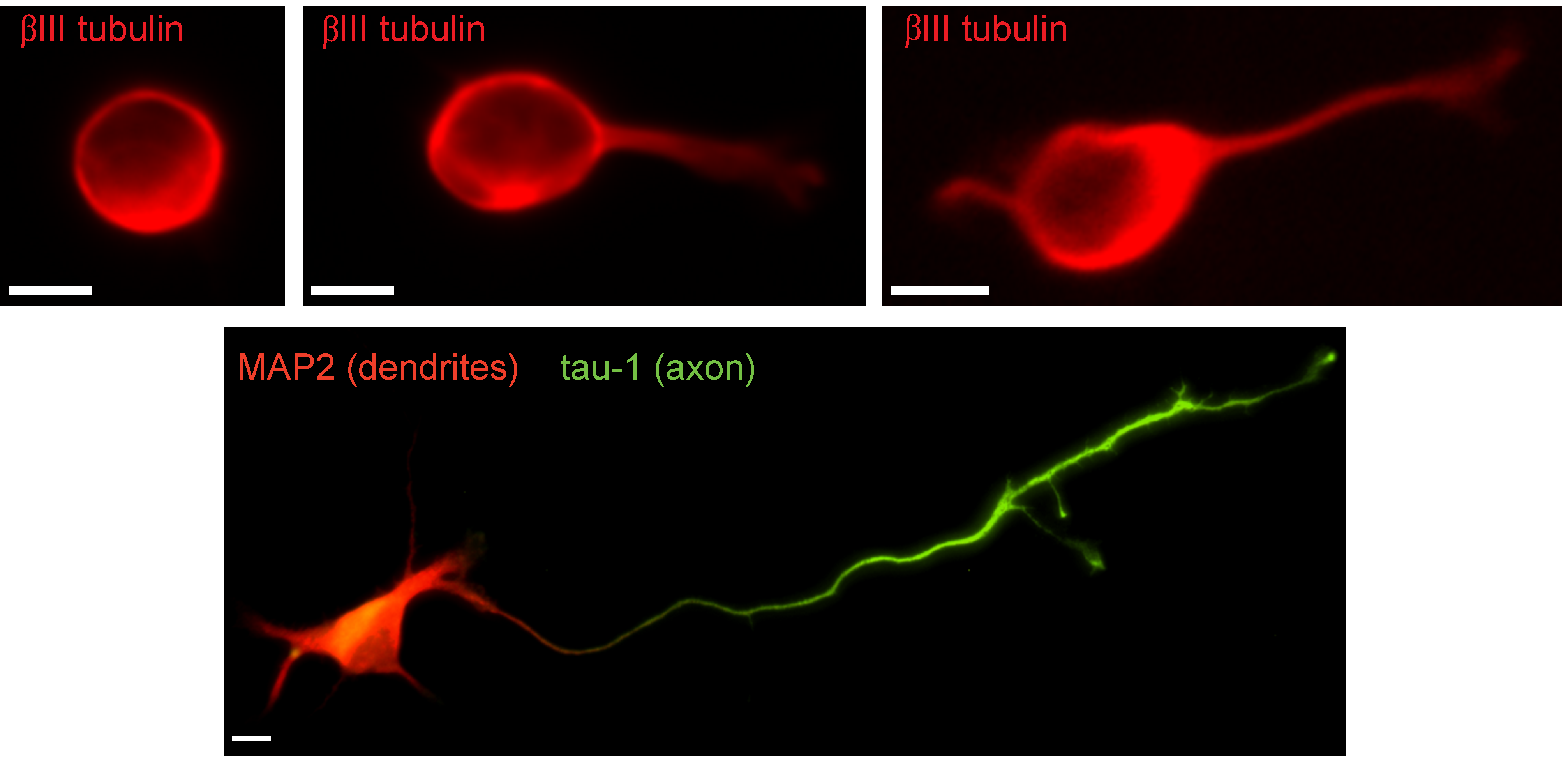

Supplement: Figure S1 — Neuron developmental stages. Hippocampal neurons were fixed at different times after plating and immunolabeled with the neuron-specific anti- tubulin antibody (red, upper three panels) or, after longer differentiation time (lower panel) with an antibody which is specific for a dendritic protein (Map2 in red) and one specific for an axonal protein (anti Tau-1 in green). (TIFF) [file pone.0024190.s001.tiff]

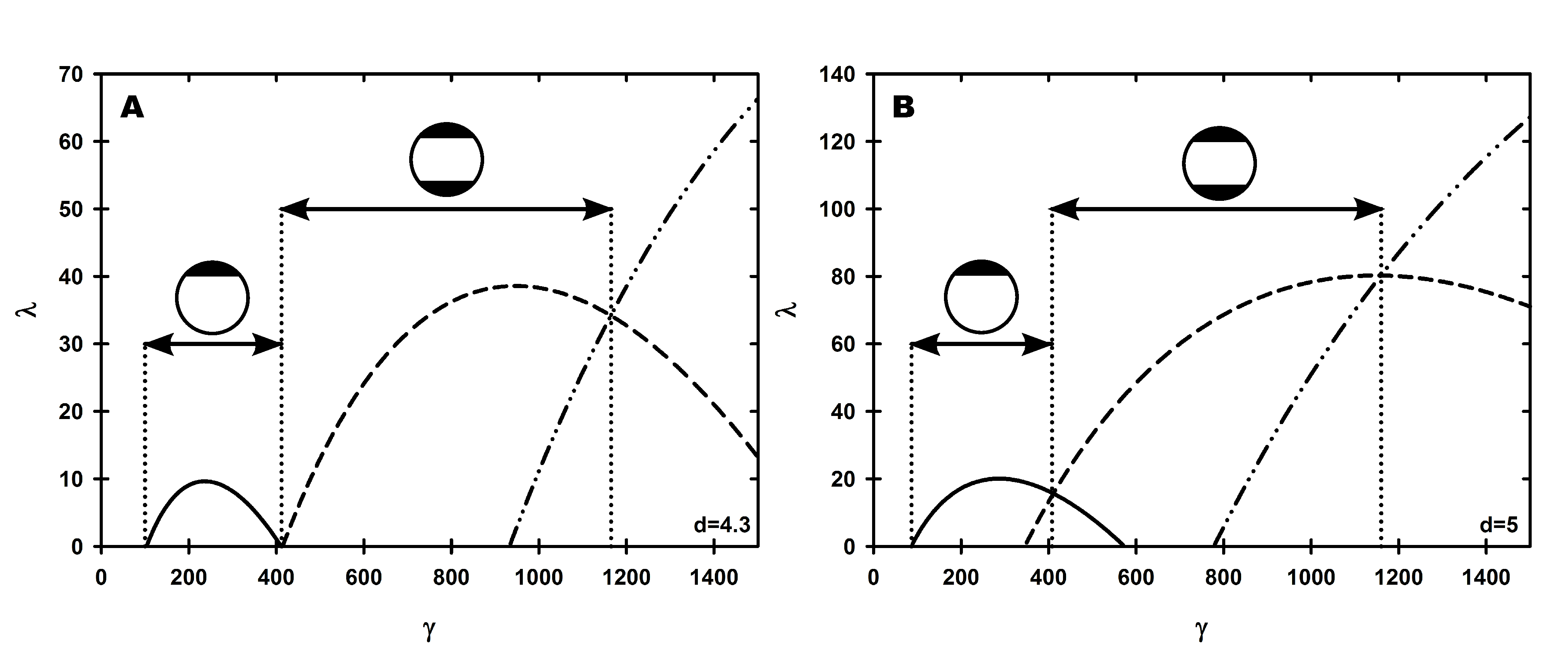

Supplement: Figure S2 — Unstable eigenvalues. The eigenvalue, , for the solution obtained linearizing Eq. (4) about the steady state versus for , (A), and , (B). Solid, dashed and dash-dotted lines represent eigenvalues for the unstable modes , and , respectively. In the regions where more than one unstable mode is present, the final number of polarity domains is defined by the highest one. For this figure , , , and . (TIFF) [file pone.0024190.s002.tiff]

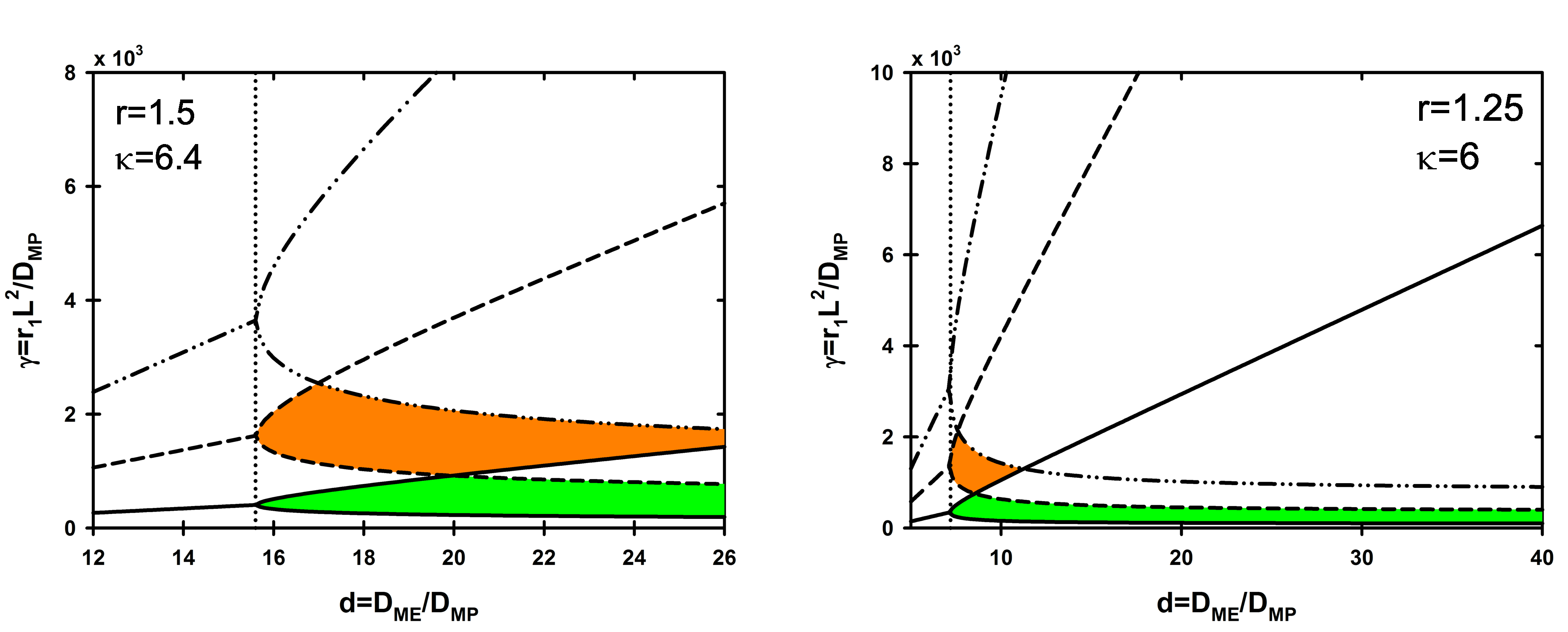

Supplement: Figure S3 — Bifurcation diagrams (I). Different bifurcation diagrams for a system characterized by Fig. 3A. On the left, and ; and on the right and . (TIFF) [file pone.0024190.s003.tiff]

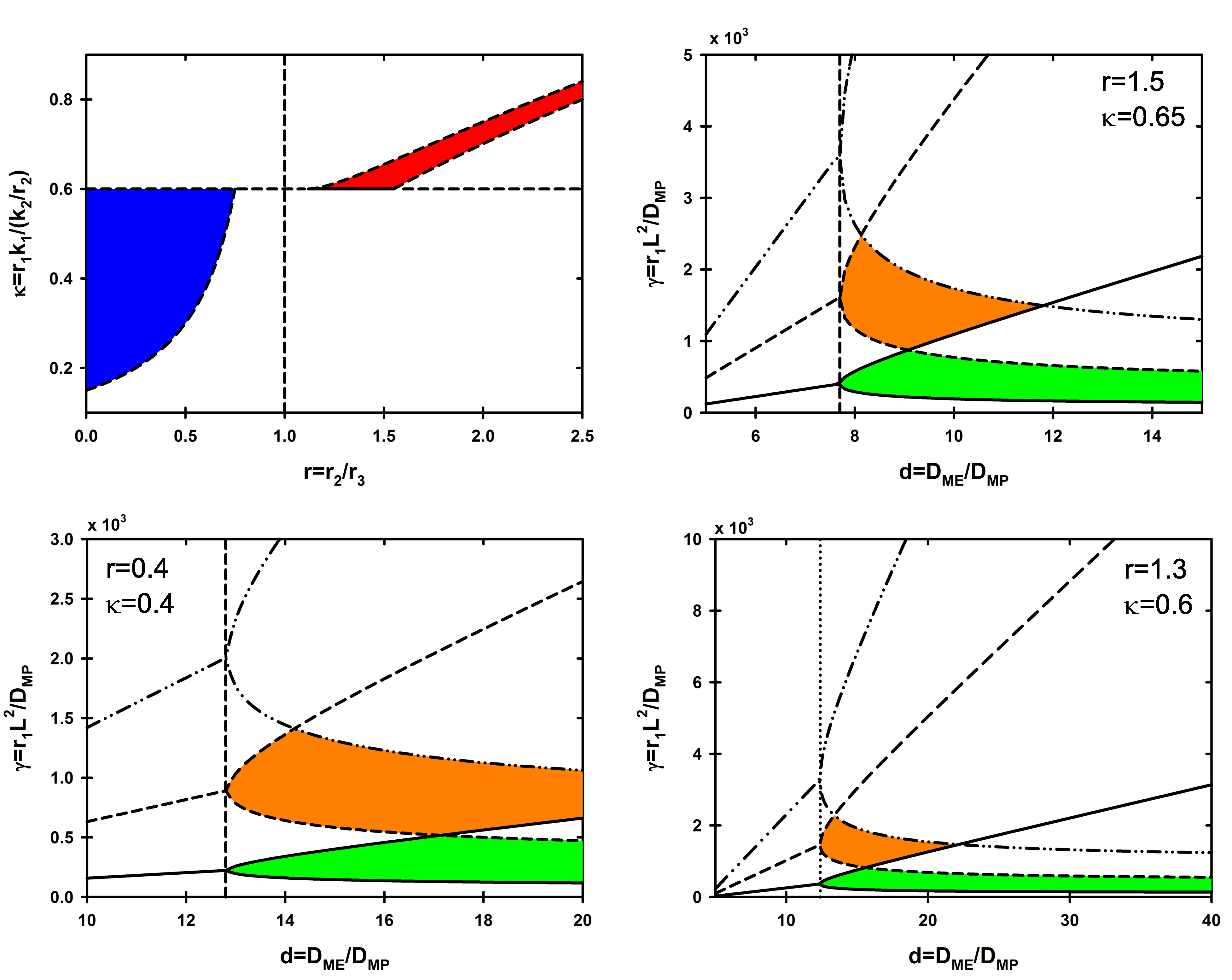

Supplement: Figure S4 — Phase and bifurcation diagrams (II). A phase diagram for a mutant phenotype with a lower modulator of endocytosis activation rate is shown in the upper left corner. Bifurcation diagrams for the indicated values are also shown. In this picture, , and , (thus, ). (TIFF) [file pone.0024190.s004.tiff]

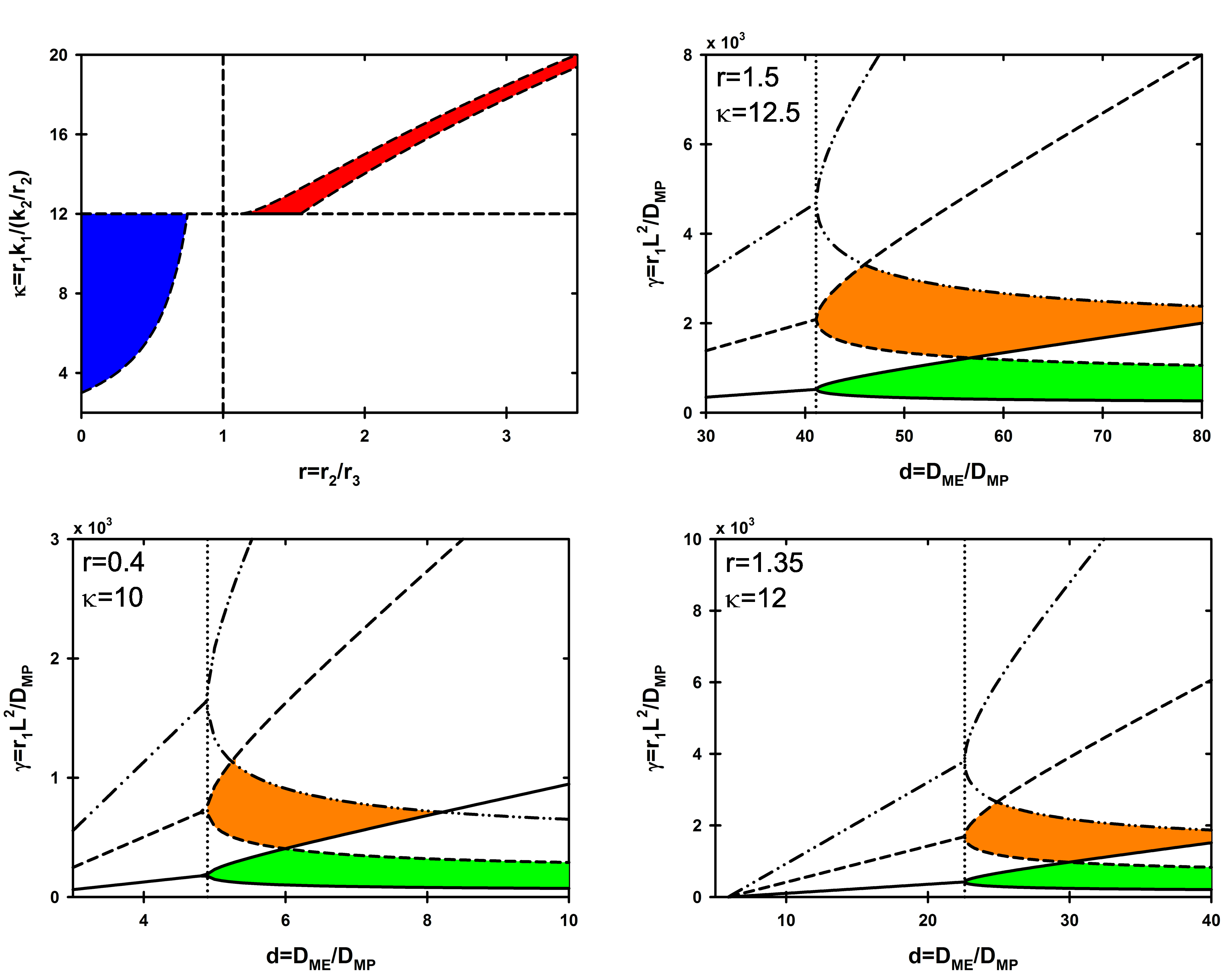

Supplement: Figure S5 — Phase and bifurcation diagrams. A phase diagram for a mutant phenotype with a higher modulator of endocytosis activation rate is shown in the upper left corner. Bifurcation diagrams for the indicated values are also shown. In this picture, , and , (thus, ). (TIFF) [file pone.0024190.s005.tiff]

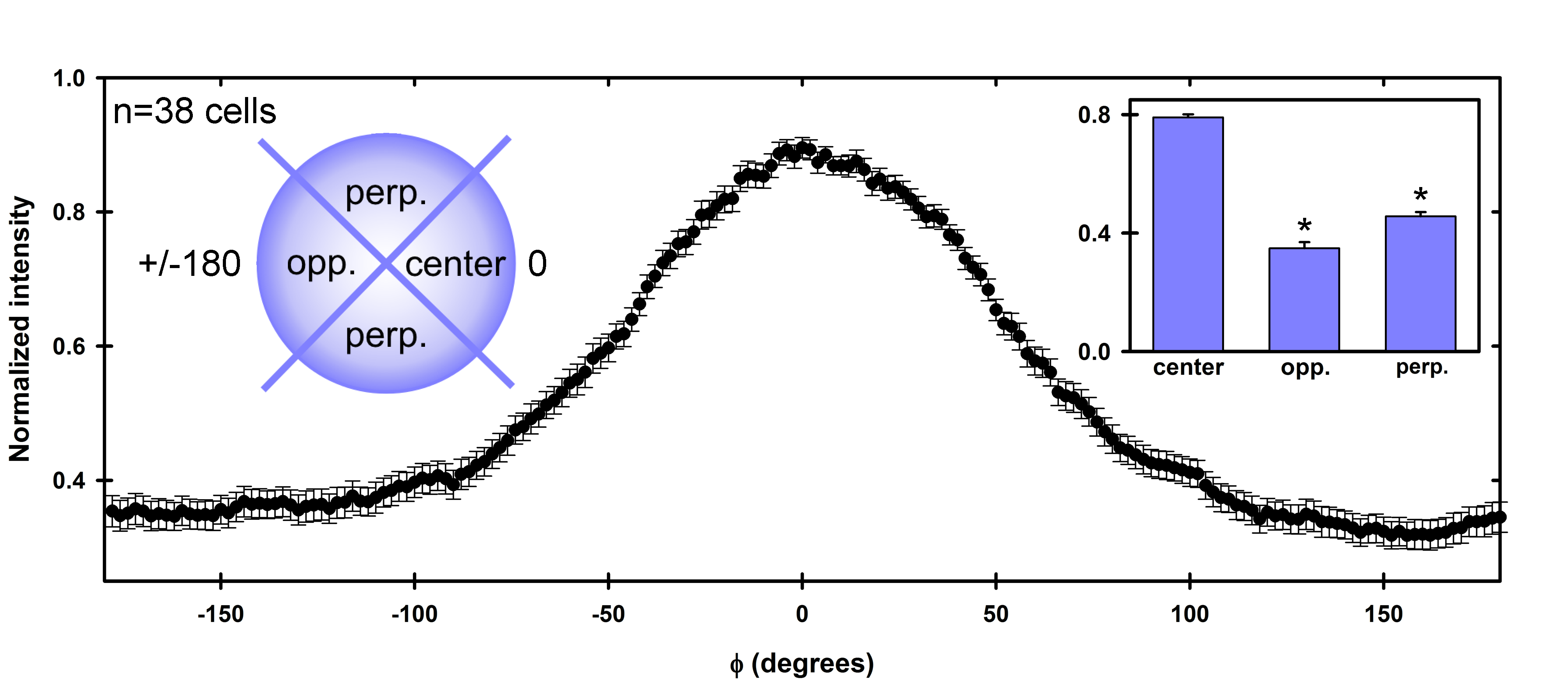

Supplement: Figure S6 — Quantification of Sec8 accumulation. Hippocampal neurons were fixed shortly after plating and immunolabeled with a neuron-specific anti- tubulin antibody and an anti Sec8 antibody. The fluorescence of Sec8 along the membrane was quantified. The signal was normalized and maxima of different cells aligned with each other respect the quarter with the highest intensity. The curve shows the mean value of 38 round neurons and the inset the analysis of the mean fluorescence of each quarter. (TIFF) [file pone.0024190.s006.tiff]

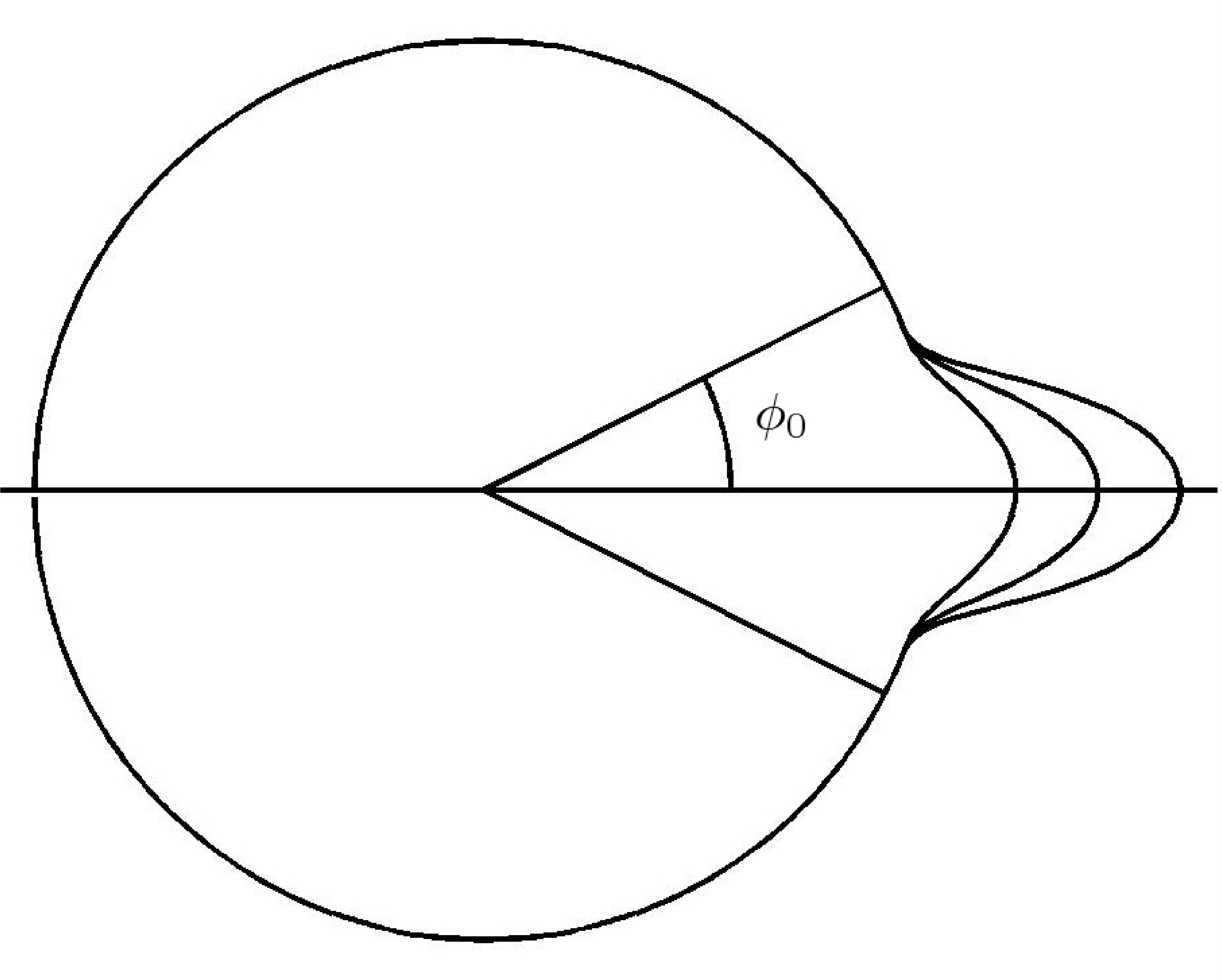

Supplement: Figure S7 — Growing domain. The curves represent the cell surface with a growing bud between the polar angles . At the cell boundary is a perfect circle. (TIFF) [file pone.0024190.s007.tiff]
